# Antifungal Susceptibility of Oral *Candida* Isolates from Mother-Infant Dyads to Nystatin, Fluconazole, and Caspofungin

**DOI:** 10.3390/jof9050580

**Published:** 2023-05-17

**Authors:** Naemah Alkhars, Anthony Gaca, Yan Zeng, Nisreen Al-Jallad, Elena Rustchenko, Tong Tong Wu, Eli Eliav, Jin Xiao

**Affiliations:** 1 Department of General Dental Practice, College of Dentistry, Health Science Center, Kuwait University, Safat 13110, Kuwait; naemah_alkhars@urmc.rochester.edu; 2 Translational Biomedical Science Program, Clinical and Translational Science Institute, School of Medicine and Dentistry, University of Rochester, Rochester, NY 14642, USA; 3 Genomic Research Center, University of Rochester, Rochester, NY 14642, USA; anthony_gaca@urmc.rochester.edu; 4 Eastman Institute for Oral Health, University of Rochester Medical Center, Rochester, NY 14620, USAnisreen_aljallad@urmc.rochester.edu (N.A.-J.);; 5 Department of Biochemistry and Biophysics, University of Rochester Medical Center, Rochester, NY 14642, USA; 6 Department of Biostatistics and Computational Biology, University of Rochester Medical Center, Rochester, NY 14642, USA

**Keywords:** antifungal agent, in vitro susceptibility, oral *Candida*, nystatin, fluconazole, caspofungin

## Abstract

The carriage of *Candida albicans* in children’s oral cavities is associated with a higher risk for early childhood caries, so controlling this fungus in early life is essential for preventing caries. In a prospective cohort of 41 mothers and their children from 0 to 2 years of age, this study addressed four main objectives: (1) Evaluate in vitro the antifungal agent susceptibility of oral *Candida* isolates from the mother-child cohort; (2) compare *Candida* susceptibility between isolates from the mothers and children; (3) assess longitudinal changes in the susceptibility of the isolates collected between 0 and 2 years; and (4) detect mutations in *C. albicans* antifungal resistance genes. Susceptibility to antifungal medications was tested by in vitro broth microdilution and expressed as the minimal inhibitory concentration (MIC). *C. albicans* clinical isolates were sequenced by whole genome sequencing, and the genes related to antifungal resistance, *ERG3*, *ERG11*, *CDR1*, *CDR2*, *MDR1*, and *FKS1*, were assessed. Four *Candida* spp. (n = 126) were isolated: *C. albicans, C. parapsilosis, C. dubliniensis*, and *C. lusitaniae*. Caspofungin was the most active drug for oral *Candida*, followed by fluconazole and nystatin. Two missense mutations in the *CDR2* gene were shared among *C. albicans* isolates resistant to nystatin. Most of the children’s *C. albicans* isolates had MIC values similar to those from their mothers, and 70% remained stable on antifungal medications from 0 to 2 years. For caspofungin, 29% of the children’s isolates showed an increase in MIC values from 0 to 2 years. Results of the longitudinal cohort indicated that clinically used oral nystatin was ineffective in reducing the carriage of *C. albicans* in children; novel antifungal regimens in infants are needed for better oral yeast control.

## 1. Introduction

Over 700 microbial species, including bacteria and fungi, inhabit the human oral cavity [1]. Colonization of the mouth by yeasts was recognized over 2000 years ago when Hippocrates reported the presence of oral thrush caused by *Candida* species in infants and people with weakened immunity [2,3]. *Candida albicans* and several related *Candida* species are opportunistic pathogens that live as benign commensal organisms in the oral cavities of healthy individuals, especially young children [4]. One reason that *C. albicans* is so successful is its ability to adapt and proliferate in a broad range of host environments, such as acidic conditions [5]. An acidic environment is detrimental to oral health because it promotes tooth cavitation. *C. albicans* can co-adhere with other commensal species, helping in biofilm formation when suitable sugar resources are available in the diet [6]. Common superficial *Candida* infections include vaginal and oral candidiasis (thrush) and chronic mucocutaneous candidiasis [7].

The literature reports that in children, the prevalence of oral thrush varies between 4% and 15% [8,9], with up to 40% in children with human immunodeficiency virus [10]. Although most cases of oral thrush are mild, the disease can spread to the lungs, pleura, or diaphragm, or even to the gastrointestinal tract or kidneys, by dissemination through the bloodstream [11]. In the past decade, cross-sectional clinical studies have observed that *C. albicans,* along with *Streptococcus mutans*, is highly abundant in the oral cavity of children with early childhood caries (ECC) [12,13]. Moreover, *C. albicans* is twice as prevalent in the biofilm of children with ECC as in caries-free children [14]. A recent systematic review and meta-analysis confirmed that the prevalence of oral *C. albicans* in children with ECC is significantly higher than in caries-free children (*p*-value < 0.01) and that children with oral *C. albicans* have 6.5 times higher odds of experiencing ECC compared to those without this yeast [15]. In keeping with these data, *C. albicans* exhibits cariogenic traits: it is acidogenic, aciduric, and able to secrete aspartyl protease, which degrades dentinal collagen [16,17]. Given the cariogenic potential of oral *Candida*, it is crucial to evaluate its profile of susceptibility to commonly available antifungal drugs, so we can develop better ways to control this yeast in the oral cavity, especially in children at high risk for ECC.

The three major classes of antifungal drugs are polyene, azole, and echinocandin [2]. Nystatin is a polyene drug that binds to the ergosterol in the cytoplasmic membrane of fungi, resulting in leakage of cell components [18]. Nystatin is not absorbed by the gastrointestinal tract; however, it has topical effects with low hepatotoxicity, no reported drug interference, and minimal adverse effects [19]. Because of the fungicidal and fungistatic properties of nystatin, it is commonly used topically for the treatment of oral candidiasis [20] and is available in suspensions, oral rinses, gels, creams, tablets, and pastilles [21]. A nystatin suspension of 100,000 IU (international units) is the treatment of choice for infants with oral thrush [22]. In fungi, the development of nystatin resistance is frequently associated with a decrease in the ergosterol content of the resistant cells [23].

Fluconazole is a systemic antifungal agent widely used in treating moderate to severe oral candidiasis [24,25]. It is fungistatic; it disrupts the synthesis of the fungal cytoplasmic membrane by inhibiting the lanosterol 14-α demethylase enzyme [26]. Fluconazole is well tolerated by patients, has mild side effects and low toxicity (although it is higher than nystatin), and is relatively inexpensive [25,27]. For children with mucosal candidiasis, the recommendation is a single daily dose of 3 mg/kg fluconazole suspension [24,28].

With the emergence of azole resistance in *Candida* species [19,29], particularly *non-albicans* [30], drugs from the new echinocandin class, such as caspofungin, are currently the first-line choice in the management of invasive candidiasis [31]. These drugs are used to treat serious fungal infections, including candidemia, esophageal candidiasis, and aspergillosis [32]. Caspofungin requires IV administration via a single daily infusion over approximately 1 h [33]. It is fungicidal, disrupting cell wall synthesis by inhibiting the enzyme 1,3-β-D glucan synthase. Echinocandins generally display superior activity against *Candida* species compared to azoles [33,34].

It is unknown whether children display a similar antifungal response to oral *Candida* as their mothers or whether, in children with recurrent colonization of *Candida*, their susceptibility profile changes over time. It is also unknown whether *Candida* becomes resistant to the drug over time. To our knowledge, no studies have compared intra- and inter-antifungal drug susceptibility between children and their mothers. Answering these questions could provide us with information about predicting the susceptibility profiles of both mothers and children. Potentially, treatment with antifungal drugs could prevent the transmission of *Candida* from mothers to their children. The present study aimed to evaluate and compare the in vitro susceptibility to nystatin, fluconazole, and caspofungin of oral *Candida* isolates collected from mothers and their children.

## 2. Materials and Methods

### 2.1. Study Design

This prospective study addressed four objectives in a cohort of mother/child dyads from birth to two years of age. We aimed to (1) assess in vitro the antifungal susceptibility of oral *Candida* isolates from the mother-child cohort; (2) compare *Candida* susceptibility between isolates from dyads of mothers and children; and (3) assess longitudinal changes in the susceptibility of the isolates from children collected between 0 and 2 years. (4) In a subset of five children who provided data through 24 months, we also evaluated the clinical effectiveness of nystatin and fluconazole in reducing infection with oral *C. albicans*. Of these five with an oral thrush diagnosis, four were treated with nystatin and one with fluconazole; none was treated with caspofungin. (5) Further explore the mutations conserved among the group of *C. albicans* isolates with nystatin resistance, non-wild-type fluconazole, and borderline high MIC values to caspofungin.

### 2.2. Study Sample

Data from pregnant women and their children were obtained from a prior birth cohort study that assessed the association between oral *Candida* in early life and the onset of dental caries in children [35]. The study protocol was approved by the University of Rochester’s Research Subject Review Board (#1248). Mother-child dyads carrying the same *Candida* species in their oral cavity were included in the antifungal susceptibility testing study (Objectives 1–3). Medical and medication information, including prior receipt of antifungal and antibiotic medications, was obtained from mothers through a self-report questionnaire and was further confirmed from participants’ medical records. For Objective 4, only children with 24 months of data and antifungal treatment were included.

### 2.3. Clinical Sample Collection and Isolate Verification

Clinical isolates of *Candida* species were obtained from the saliva and dental plaque of pregnant women and their children. For saliva sample collection, mothers were instructed to provide unstimulated saliva by spitting it into a sterilized 50 mL centrifuge tube and were asked not to brush their teeth for 2 h before sample collection [36,37]. Meanwhile, for children, the SalivaBio Infant’s Swab (SIS) (Item No. 5001.08) (Salimetrics, Inc., Carlsbad, CA, USA) was used to collect saliva samples [38]. A sterilized periodontal scaler was used to collect plaque from all tooth surfaces [36,37]. Isolate types were identified by specific color on tests using BBL™ CHROMagar™ Candida (BD, Sparks, MD, USA) and further verified with whole genome sequencing (WGS). Cells from −80 °C stock cultures were streaked on Yeast Peptones Dextrose agar (YPD) and incubated at 37 °C for 20–24 h.

### 2.4. DNA Extraction and Library Preparation for Whole Genome Sequencing

As a second and more reliable step in the verification of the *Candida* isolates, whole genome DNA (gDNA) was extracted from the clinical isolates using the MasterPure™ kit (Lucigen Corp., Middleton, WI, USA) following the manufacturer’s instructions. Libraries were constructed using the Nextera XT kit (Illumina, Inc., San Diego, CA, USA) with 3 ng of gDNA as input. Fragment size profiles and quantification of the libraries were measured using the Fragment Analyzer and Qubit, respectively. The libraries were normalized to 1.75 nM and sequenced on NovaSeq 6000 using a SP flow cell with 150-bp paired-end reads. Sequence analysis was performed for the whole gene sequences of the genes associated with antifungal resistance, *ERG3*, *ERG11*, *CDR1*, *CDR2*, *MDR1*, and *GSC1* (also known as *FKS1*).

### 2.5. Laboratory Preparation of Antifungal Drugs

*Candida* isolates were tested against nystatin (VWR International, LLC, Bridgeport, NJ, USA), fluconazole, and caspofungin (APExBIO, Houston, TX, USA). The drugs were dissolved in water or dimethyl sulfoxide (DMSO, Alfa Aesar, Ward Hill, MA, USA) according to the manufacturer’s instructions. A stock drug solution was prepared that was 100 times higher than the desired testing concentration, or at least 1.28 mg/mL.

### 2.6. In Vitro Antifungal Drug Susceptibility Testing

Antifungal susceptibility testing was performed using the broth microdilution method according to the Clinical and Laboratory Standards Institute (CLSI) M27-A3 [39] with modification, using RPMI 1640 growth medium (0.2% glucose with glutamine and without bicarbonate) (Gibco™, Thermo Fisher Scientific, Waltham, MA, USA). The medium was buffered with 3-(N-morpholino) propanesulfonic acid (MOPS, Sigma-Aldrich, St. Louis, MO, USA) at a pH of 7. Yeast inoculums were prepared in 5 mL of RPMI 1640 medium, and the cell density in the resulting suspension was adjusted to 0.5 McFarland standard, corresponding to a stock inoculum of 1–5 × 10^6^ CFU/mL (colony-forming units per milliliter). The suspension was diluted to obtain a final working cell density of 1–5 × 10^3^ CFU/mL. For each drug, two-fold serial dilutions were prepared, yielding final concentrations ranging from 16 to 0.03 μg/mL for nystatin, 64 to 0.125 μg/mL for fluconazole, and 8 to 0.015 μg/mL for caspofungin. Subsequently, 100 µL of the working inoculum solution and 100 µL of the diluted antifungal solution were seeded into a sterile, disposable, flat-bottomed 96-well plate (Greiner Bio-One, Monroe, NC, USA). In each well, the final inoculum density was 0.5–2.5 × 10^3^ CFU/mL. The plates were incubated in ambient air without agitation at 35 °C for 24 to 48 h, and the cell densities were noted visually. *C. albicans SC5314* or *C. krusei* ATCC 6258 were used as references for quality control in all the experiments. To ensure rigorous and reproducible data, each experiment was performed in technical triplicate.

### 2.7. Spectrophotometric Measurement and Determination of Minimum Inhibitory Concentration (MIC) of Antifungal Drugs

To eliminate the subjective determination of MIC values by visual inspection, spectrophotometer readings were carried out according to EUCAST guidelines. The MIC for fluconazole and caspofungin was defined as the lowest drug concentration that resulted in 50% growth inhibition compared to that of the growth control wells. For nystatin, the MIC was defined as the lowest drug concentration that resulted in 90% growth inhibition compared to that of the growth control wells [40]. The plates were read spectrophotometrically at 600 nm absorbance after 24 and 48 h using a microtiter plate reader, the Tecan Infinite M200 PRO (Tecan, Männedorf, Switzerland). The MIC values and range were calculated. The isolates were classified as susceptible (S), intermediate (I), susceptible dose-dependent (SDD), and resistant (R), based on clinical breakpoints (CBP) recommended in the documents M27-A3 and M27-S4 [39] and expressed visually as heatmaps (Figure S4). However, polyenes, including nystatin, lack defined CBPs. To facilitate comparisons with other antifungal drugs, epidemiological cutoff values (ECVs) of amphotericin B (AMB, 2 μg/mL) were selected for all isolates [41]. Moreover, because no defined CBPs were available for *C. dubliniensis* and *C. lusitaniae*, ECVs were used to separate susceptible and resistant isolates.

The antifungal susceptibility of isolates from mothers, compared to those of isolates from their respective children, was determined, as was the MIC change over time for children.

### 2.8. Bioinformatic Analysis of Antifungal Medication Resistance Genes

Pre-processed reads (adapters and low-quality bases removed) were mapped to the reference genome of *C. albicans SC5314* with the assembly accession reference sequence GCA_000784635.1 using BWA MEM (v 0.7.17) [42]. The output SAM alignment file was converted to a sorted BAM file using samtools (v1.9) [43]. Variant call files (VCFs) were generated from the sorted BAMs using bcftools mpileup (v1.4) [43] with a minimum mapping quality score of 5. The VCFs were further filtered to exclude variants with quality scores of <20 and coverage depths < 10. Variants were annotated with snpEff [44] (v4.3t) using the pre-compiled *C. albicans SC5314* genome. Annotated VCF files were further filtered using the bcftools filter (v1.14; [43]) with the option—regions-file to include only genomic regions pertaining to 6 antifungal genes of interest. Furthermore, the bcftools isec (v1.14 [43]) was used to identify variants common or unique to different groups of *C. albicans* strains. For each drug, 2 groups were made for the comparison as follows: nystatin resistant vs. susceptible, fluconazole wild type (WT; MIC ≤ 0.5 μg/mL) vs. non-wild type (NWT; MIC > 0.5 μg/mL), and caspofungin normal MIC values (MIC < 0.25 μg/mL) vs. borderline high MIC values (MIC = 0.25 μg/mL). The isolate is likely to be WT and should respond to treatment with that drug if the MIC falls below the ECV for a particular species of fungus. However, if the MIC is above the ECV, then the isolate is likely NWT and may have acquired resistance to the drug [41,45]. A set of conserved variants across all isolates of a given group was constructed using bcftools isec with the—nfiles option set to the number of isolates (individual VCF files) being compared. The resulting VCF file containing the conserved variants across the resistance group was then used as input to bcftools isec to identify a unique variant signature for each group.

### 2.9. Data and Statistical Analysis

The data were analyzed using SAS (SAS Institute) and displayed using GraphPad Prism version 9.4.1. Categorical variables were expressed as frequencies (%) and analyzed using Pearson’s chi-square test or Fisher’s exact test. The unpaired *t*-test and Mann–Whitney U test were used to compare differences in MICs among the mother/child isolates. Multiple logistic regression was used to assess factors associated with nystatin resistance (Y/N) in *C. albicans* isolated from mothers and children. Factors included in the regression were race, ethnicity, gender, and prior clinical antifungal medication consumption. Differences were considered significant at a *p*-value < 0.05.

## 3. Results

A total of 41 mother-child dyads in which the same type of oral *Candida* was detected were included in the study. The demographic, socioeconomic, medical, and oral characteristics of mothers and their children are shown in Table 1 and Table S1. The participating mothers were 26.4 ± 5.5 years old. Among the young children, 53.7% were female. With respect to race, about 67% of the dyads were black, and 20% were white. Approximately 10% of mothers and 5% of children were of other or mixed races. The majority of the dyads were non-Hispanic. Half of the mothers in the study were diagnosed with emotional conditions such as depression or anxiety by their physicians. These emotional conditions have been found to potentially compromise the immune system and increase the risk and severity of fungal infections [46], such as oral candidiasis. Approximately one-fifth of the children were delivered by C-section (22%). Regarding oral health conditions, most mothers had dental caries (78%), whereas 10 children had ECC by two years of age (33.3%). Diaper rash in children was more prevalent than oral thrush (39% versus 19.5%). Children’s daycare attendance was low and remained below 20% throughout the period from birth to two years. Nearly all mothers were care providers for their children, whereas a range of 39–62% of fathers were involved in caring for their child between birth and two years.

The feeding patterns, including breastfeeding (exclusively), bottle feeding (exclusively), combined breast and bottle feeding, night feeding, and consumption of solid food, are illustrated in Figure S1. Exclusive breastfeeding gradually decreased from 32% at one month to 0% at 18 months. On the other hand, exclusive bottle feeding nearly doubled from 36% at one month to 61% at six months. It remained stable between six and 12 months and sharply decreased at 18 months. The number of children who had night bottle feeding was high (72%) during the first six months, with a sharp drop after six months, reaching 3% at the age of two years. Consumption of solid food started as early as two months and reached 100% at 12 months.

### 3.1. MIC of Clinical Candida Isolates

A total of 126 clinical *Candida* isolates from saliva (123) and plaque (3) were examined; these included 114 *C. albicans*, six *Candida parapsilosis*, four *Candida dubliniensis*, and two *Candida lusitaniae*. All isolates were confirmed by WGS; sequence reads were deposited in the NCBI Sequence Read Archive (SRA: Bioproject number PRJNA926612). The in vitro susceptibility of clinically isolated *Candida* species to nystatin, fluconazole, and caspofungin is shown in Table 2. Each species of *Candida* demonstrated a somewhat different range of susceptibility to the three drugs tested.

### 3.2. Isolate Susceptibilities to Antifungal Drugs

Classification of isolate susceptibilities to the tested antifungal drugs was based on clinical breakpoints (CBP) recommended in CLSI documents [47], as shown in Table 3, Table 4 and Table 5 and Tables S2–S6. CBP is the MIC value that is used to categorize the susceptibility of an isolate (S, SDD, I, or R). To date, no CBPs or ECVs for nystatin have been defined. For this study, to allow comparison with other antifungal drugs, ECVs of amphotericin B (AMB, 2 μg/mL) were selected for all species [41].

Most drugs were active against oral *Candida* isolates. Caspofungin was the most active drug; all isolates were susceptible to this drug. *C. parapsilosis* isolates (33.3%) fell into the SDD category for fluconazole. The overall resistance rate to nystatin was 5.6% (7 out of 126 isolates). Prenatal and child-isolated resistance to nystatin was associated with maternal smoking (Fisher exact test; *p*-value = 0.027). Moreover, child isolate resistance to nystatin was associated with six months of solid food intake (Fisher exact test; *p*-value = 0.012) and 18 months of carriage of *C. krusei* in plaque (Fisher exact test; *p*-value = 0.005). In addition, isolate resistance to nystatin was related to the child’s experience of two prior episodes of otitis media and two periods of treatment with oral amoxicillin (Fisher exact test; *p*-value = 0.027). Resistance to nystatin was not associated with a history of yeast infection or antifungal treatment (Fisher exact test; *p*-value = 0.57).

### 3.3. Candida MIC Distribution among Mother-Child Dyads

**Figure 1:**
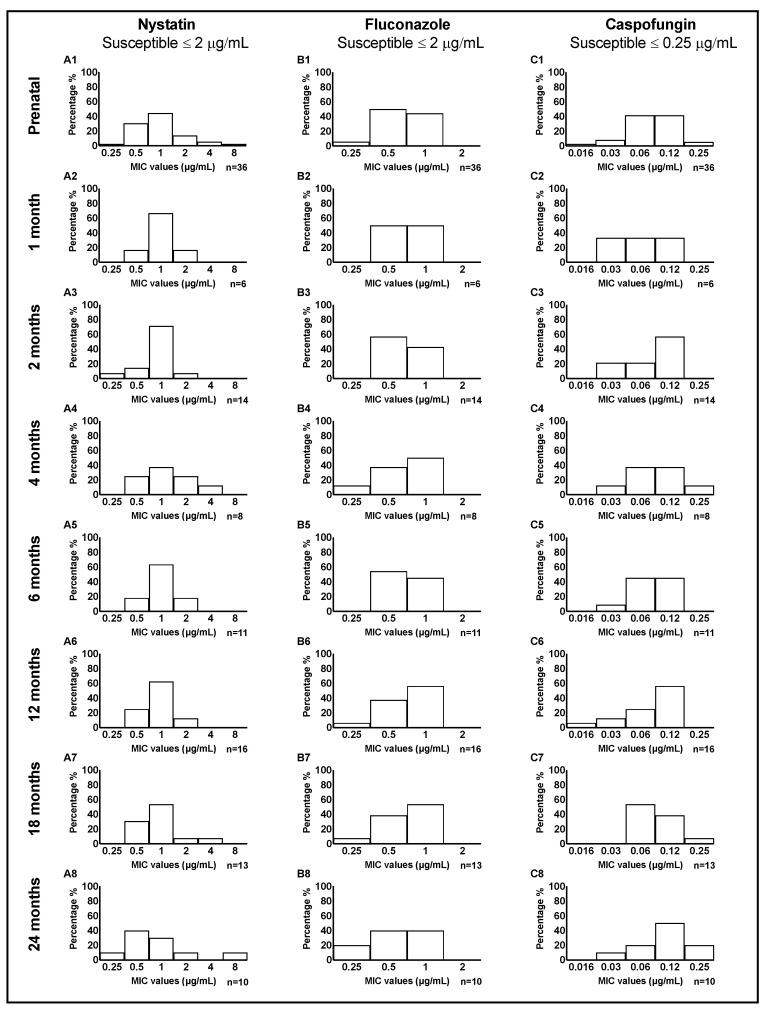
Distribution of MIC values of three antifungal medications tested in 114 *C. albicans* clinical isolates from mother-child dyads. MIC values (averaged for triplicate measurements) are illustrated separately for nystatin, fluconazole, and caspofungin. For nystatin (**A1–8**), the MIC of most of the clinical isolates clustered around 1 μg/mL; a higher MIC value (4–8 μg/mL) was seen in 8% of isolates from mothers and 4% of isolates from children. Regarding fluconazole (**B1–8**), mothers and children had the same frequency distribution of MIC values, centered around 0.5–1 μg/mL at almost all child ages. For caspofungin (**C1–8**), 42% of the mothers had a MIC value of 0.06, and the same percentage had a MIC value of 0.12 μg/mL. In early infancy, most children had a low MIC value, with a shift towards higher values of 0.12 and 0.25 μg/mL at 24 months.

The distribution of MIC values for nystatin, fluconazole, and caspofungin in a total of 114 *C. albicans* clinical isolates from mother-child dyads is illustrated in Figure 1. Overall, for nystatin and fluconazole, we observed a somewhat similar distribution of MIC values between mothers and children. For caspofungin, at 24 months, we noticed more isolates with higher MIC values of 0.12 and 0.25 μg/mL. The distribution of MIC values tested in four *C. dubliniensis* and six *C. parapsilosis isolates* from mother-child dyads is illustrated in Figures S2 and S3, respectively.

### 3.4. Comparison of Antifungal Susceptibility between Mothers and Children

**Figure 2:**
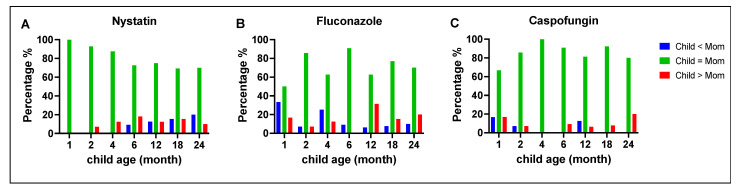
Comparison between clinical isolates with mothers and children of *C. albicans* susceptibility to antifungal medications. The MIC values (averaged for triplicate measurements) of *C. albicans* for nystatin (**A**), fluconazole (**B**), and caspofungin (**C**) were compared between mothers and children at different time points. For nystatin, a high similarity percentage was observed, ranging between 70 and 100% (**A**). For fluconazole, the similar percentage ranged from 50 to 90%, with a higher proportion of children having high MIC values at 12 and 24 months (**B**). For caspofungin, at one month of age, 17% of the children’s isolates had lower MIC values than the isolates from their mothers, which reduced to 0% at 24 months. In contrast, the proportion of children with higher MIC values increased from 17% at one month to 20% at 24 months (**C**).

*C. albicans* MIC values between mothers and children were compared at different time points, as illustrated in Figure 2. Overall, the majority of the children’s *C. albicans* isolates had MIC values that were similar to strains isolated from their mothers (green bars). Notably, for fluconazole, at 12 months, 31% of the children’s *C. albicans* isolates had MIC values that were higher than the isolates from their mothers (red bars). For caspofungin, at 24 months, 20% of the children’s *C. albicans* isolates had MIC values that were higher than the isolates from their mothers (red bars).

When we compared the MIC values of the three *C. parapsilosis* dyads for nystatin and fluconazole, we found a different pattern: an equal proportion of those with the same, high, or low MIC values (each 33.33%). For caspofungin, two dyads had the same MIC values, and in the third dyad, the child isolate had a lower MIC value than the mother.

When we compared the two *C. dubliniensis* dyads, we found that the MIC values were the same for mothers and children for nystatin and fluconazole. For caspofungin, one dyad had the same MIC values; in the second dyad, the mother’s strain had a higher MIC value than the child’s. Finally, for *C. lusitaniae*, the single dyad had the same MIC values for nystatin and fluconazole, but for caspofungin, the child’s isolate had a higher MIC value than the mother’s.

### 3.5. Changes of Antifungal Medication Susceptibility in Early Life

**Figure 3:**
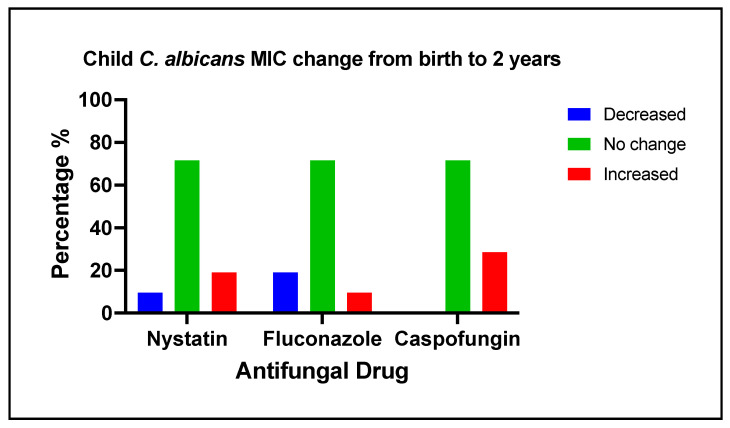
Changes in *C. albicans* MIC to antifungal medications among 21 children in early life. The changes in *C. albicans* MIC values (averaged for triplicate measurements) to antifungal medications among children from birth to two years of age are categorized as decreased, no change, or increased. Approximately 70% of the clinical isolates from children did not have changed MIC values for nystatin, fluconazole, or caspofungin. For nystatin, 19% of the clinical isolates had an increased MIC, and 14% had decreased MIC values over time. For fluconazole, 10% of the clinical isolates had an increase, whereas 19% had a decrease in their MIC values over time. Notably, for caspofungin, the MIC values increased in 29% of the isolates.

The changes in *C. albicans* MIC values to antifungal medications among 21 children from birth to two years of age are categorized as decreased, no change, or increased, as shown in Figure 3. Nystatin MIC value increase was associated with maternal hypertension (Fisher exact test; *p*-value = 0.029). Fluconazole MIC value increase was associated with 6 months of solid food intake (Fisher exact test; *p*-value = 0.029) and 18 months of sweet index (Fisher exact test; *p*-value = 0.019). Caspofungin MIC value increase was associated with mother hypertension (Fisher exact test; *p*-value = 0.048), mother plaque *C. albicans* detection (Fisher exact test; *p*-value = 0.012), 4 and 6 months breastfeeding and six months night bottle feeding (Fisher exact test; *p*-value = 0.048), 18 months *C. krusei* carriage, and having multiple vomiting, diarrhea, and constipation (Fisher exact test; *p*-value = 0.035). No factors were found to be associated with a decrease in the isolates’ MIC values.

### 3.6. Effectiveness of Clinical Oral Consumption of Antifungal Medication in Early Life

**Figure 4:**
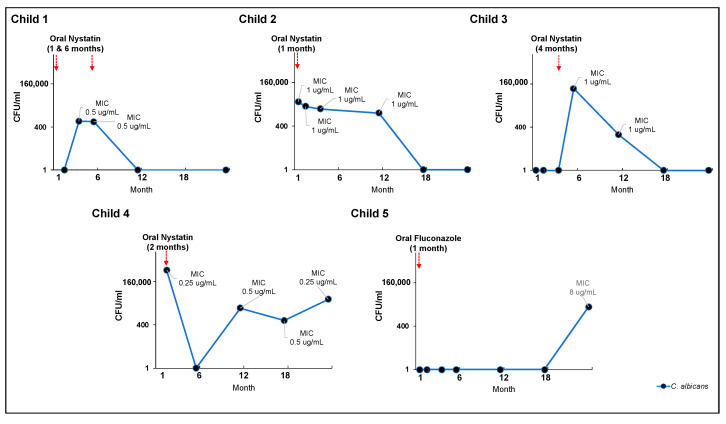
*C. albicans* carriage level and oral antifungal treatment (nystatin or fluconazole). CFU—colony forming unit; MIC—minimum inhibitory concentration. Values averaged for triplicate measurements. Five children completed the 24-month visit and received oral antifungal treatment (nystatin or fluconazole). The majority of antifungal drugs were taken between the ages of one and six months. At 24 months, 3 children were negative for *C. albicans* in their saliva, while 2 remained positive. Despite treatment with oral antifungal medication, their *C. albicans* salivary carriage was still >400 CFU/mL, the cut-off value for diagnosing oral candidiasis [49]. Notably, child 5 was given fluconazole at the age of 1 month, which resulted in a high nystatin MIC value for *C. albicans* by the time he was 24 months old.

A total of 5 children had oral antifungal treatment (either nystatin or fluconazole) and completed the 24-month visit as shown in Figure 4. Most of the antifungal medications were taken in early life, between one and six months. At 24 months, three children no longer had detectable salivary *C. albicans,* while two were still positive. Their *C. albicans* salivary carriage was still >400 CFU/mL, the cut-off value for the diagnosis of oral candidiasis [49], despite treatment with oral antifungal medication. At 24 months, it was observed that early administration of fluconazole to child 5 led to a high nystatin MIC value for *C. albicans*.

### 3.7. Point Mutations in Antifungal Resistance Genes

The 6 *C. albicans* strains that were resistant to nystatin exhibited two missense mutations in the *CDR2* gene and 28 synonymous mutations when compared to the *SC5314* reference sequences (Table 6 and Table S7). The nystatin-susceptible isolates did not show any shared mutations. However, some isolates did carry mutations found in the resistant group. For fluconazole, no mutations were conserved among NWT isolates, and five synonymous mutations were shared among the WT isolates (Table S8). Four missense mutations in three genes and 29 synonymous mutations were identified in the seven isolates with a caspofungin borderline high MIC value of 0.25 μg/mL (Table 6 and Table S9). The *FKS1* gene mutation was unique to this group of isolates and was not observed in any isolate with a MIC value of less than 0.25 μg/mL.

## 4. Discussion

Our in vitro study showed that caspofungin was the most effective antifungal drug against all the tested *Candida* isolates, followed by fluconazole and nystatin. Resistance to nystatin was observed in 5.6% of the isolates. Although most of the children’s *C. albicans* isolates had MIC values similar to those of the strains isolated from their mothers, the children also carried strains that were less susceptible to antifungal drugs than the maternal isolates. In nystatin-resistant isolates, two missense mutations in the *CDR2* gene were identified.

Our finding that *Candida* was resistant to nystatin in 5.6% of the samples requires interpretation. Since CBP and ECV cut-offs have not been defined for nystatin, we used the ECV of amphotericin B instead because both drugs have the same mechanism of action. Sutton et al. also used a value > 2 μg/mL as an endpoint to determine resistance to nystatin, and their finding of *C. albicans* having the highest level of resistance compared to other strains agrees with our results. Moreover, De-Ia-Torre et al. and Zida et al. reported nystatin resistance in 5% and 6.3% of the isolates tested, respectively [27,50]. Our findings further confirm that some *Candida* strains can display resistance to nystatin (although at a relatively low percentage). Results from our longitudinal cohort (Figure 4) indicated that clinical use of oral nystatin was ineffective in eliminating or reducing the carriage of *C. albicans* in children’s oral cavities. Although oral nystatin was prescribed for seven children, *C. albicans* salivary carriage remained higher than 400 CFU/mL after treatment, a value consistent with a laboratory diagnosis of oral candidiasis [49].

Previous research has demonstrated that nystatin is less effective than oral fluconazole or miconazole gel in treating oral thrush in immunocompetent infants [28,51]. This finding is supported by our in vitro results. However, in our study, only one child received oral fluconazole treatment while seven received oral nystatin treatment, making it difficult to compare the clinical efficacy of those two medications. Moreover, although we found that children’s isolates of *C. albicans* were not more susceptible to antifungals than adult isolates, pediatric antifungal medication contains only one-sixth of the dose used for adult antifungal medication. This difference could potentially explain the frequent failure of antifungal therapy in the children studied.

Our in vitro data from Objectives 1, 2, and 3 reveal that none of the tested isolates were resistant to fluconazole, a finding similar to that of Brito et al. [45], who examined *Candida* species oral isolates from HIV and control groups, and Jewtuchowicz et al. [52,53], who studied *Candida* species in healthy subjects with periodontal disease. In contrast, Zida et al. reported the highest resistance to azole, particularly fluconazole (66.5%), with a drug resistance rate of 72.3% for *Candida* isolates from a vulvovaginal source and 59.0% for isolates from an oral source [50]. *C. krusei* is inherently resistant to fluconazole [47,54]. These differences from our results could be attributed to the differences in the *Candida* species studied. In our mother-child pairs, we did not detect *C. krusei*.

In our study, although none of the subjects were exposed to caspofungin, we saw an increase in the MIC value of caspofungin in 29% of the children’s isolates. This increase in the MIC value may suggest the development of a more drug-tolerant strain in children who harbor *C. albicans* for extended periods. According to recent reports on mucosal candidiasis, caspofungin treatment was not superior to fluconazole, and there was concern about relapse and reinfection after caspofungin treatment [33]. These findings emphasize the importance of developing a new antifungal medication with superior activity.

In our study, two missense mutations in the *CDR2* gene, which codes for efflux pump proteins, were identified in all strains with MIC values above the ECV. However, those mutations were also found in some susceptible isolates, and casual inferences could not be drawn. Polyene resistance has been linked to a reduction in the ergosterol content of the plasma membrane [55]. More research is needed to quantify membrane ergosterol levels and confirm the link between ergosterol content and resistance level. Furthermore, our findings failed to identify mutations specific to fluconazole NWT *C. albicans* strains. Mechanisms of fluconazole resistance have been attributed to an alteration in *ERG3/ERG11* and/or *CDR*/*MDR* genes [33]. Moreover, several point mutations in the *FKS1* subunit of glucan synthase have been identified and linked to echinocandin resistance [56]. Caspofungin resistance was not observed in our study; however, isolates with borderline high MIC values were grouped together, and four missense mutations were identified, one of which was in the target gene *FKS1* and unique to those isolates.

Our finding of no cross-resistance between fluconazole, nystatin, and caspofungin confirms previous studies and supports the view that no cross-resistance occurs between the classes of azoles, polyenes, and echinocandins [29,50,57]. This finding suggests that if an isolate is resistant to one drug, it may be vulnerable to another. However, De-Ia-Torre et al. reported that multiple isolates exhibited cross-resistance or a combination of resistance in non-wild type (NWT) strains to different drugs (fluconazole, nystatin, itraconazole, posaconazole, amphotericin B, and voriconazole) [27]. Further study is needed to resolve this discrepancy.

We found that the susceptibility of *Candida* isolates in children who received oral antifungals for all tested drugs did not differ from isolates taken from those who received no oral antifungals. In this small sample, logistic regression did not identify significant factors associated with *C. albicans* resistance to nystatin or child MIC increases for nystatin, fluconazole, or caspofungin. Some studies have reported a correlation between antifungal use and resistance, but isolates in those studies were obtained from subjects with repeated or long-term antifungal use or children given antifungal prophylaxis due to immunosuppression [58,59]. Our subjects, in contrast, were healthy with no immunosuppression or long-term antifungal use.

Even though our laboratory results indicated that *C. albicans* was susceptible to antifungal medication, we still observed treatment failure. A lack of treatment compliance, incorrect dosage, or frequency could all be possible explanations. Nystatin should be given four times per day for 10–14 days for infants and children with oral candidiasis [60]. Our findings are in line with previous clinical studies indicating that mycological cure rates are low after nystatin treatment, ranging between 5 and 11% [10,28,61]. Although in vitro antifungal susceptibility testing could be a useful tool for predicting therapeutic outcomes, it does not always accurately reflect what occurs in vivo due to individual natural variability, drug properties, and the differential characteristics of microorganisms in each individual [18,62].

## 5. Limitations and Alternative Approach

Our study is limited by a relatively small sample size and a population from a single site in one US city, so the results may not generalize to other populations. In addition, only oral samples were collected and assessed, not samples from other body parts that can harbor *Candida*. Objective 4 was a preliminary investigation with only 5 children without a systematic treatment protocol. Moreover, the study was in vitro, and the bioavailability of antifungal drugs can vary depending on several factors, such as age, sex, body weight, and metabolism [63]. In vitro studies may not accurately reflect outcomes in vivo due to differences in physiological processes [64]. Therefore, it is important to conduct clinical studies in the intended population, such as mothers and children, to obtain accurate data on drug safety and efficacy. This data can help healthcare providers make informed decisions on treatment options.

In addition, our in vitro broth microdilution method was performed on planktonic *Candida,* which does not mimic the biofilm nature of *Candida* in the oral cavity. The experiment could have been performed after *Candida* biofilm formation in the 96-well microtiter plates. Alternatively, we could have tested the effect of the drugs on the duo-species biofilm of *S. mutans* and *C. albicans* using the biofilm model. The latter method is helpful in allowing direct visualization and quantification of the extrapolysaccharide (EPS) and bacterial cells within intact biofilms, and it allows measurement of dry weight, total protein, and polysaccharide composition. We chose our method because it is the gold standard for antifungal susceptibility testing and thus could provide a foundation for future experiments.

## 6. Conclusions

Despite the fact that nystatin is currently the recommended drug for the treatment of infants with oral candidiasis, its in vitro efficacy is inferior to fluconazole and caspofungin. Our findings highlight the existence of *Candida* strains that are not susceptible to nystatin, as confirmed by laboratory *Candida* detection after nystatin treatment. The implications of the preliminary results highlight the need for a drug other than nystatin for the treatment of infants with oral candidiasis. To determine the burden of infections caused by antifungal-resistant *Candida* strains, we suggest using proactive surveillance methods to evaluate resistance development in *Candida* infections. The high activity of fluconazole and lack of in vitro resistance of isolates to this drug may suggest the value of continuing to use this medication for the treatment of oral candidiasis and, potentially, dental caries.

The majority of the children’s *C. albicans* isolates from birth to two years old had similar MIC values to the strains isolated from their mothers, although children also carry strains that are less susceptible to antifungal drugs than those from their mothers. This could explain treatment failure and suggest that a different dosage is required to more effectively treat children’s *Candida* infections. The increase in caspofungin MIC values in children’s *C. albicans* may indicate decreased activity of this antifungal medication in individuals who have carried *C. albicans* for a long time. Given that caspofungin is not appropriate for mild disease because it requires intravenous administration, it would be advisable to identify some alternative antifungal agents as an adjunct for the therapeutic prevention of oral diseases.

## Figures and Tables

**Table 1:** Demographic, socioeconomic, medical, and oral characteristics of mother-child dyads (n = 41).

Categories		Mother n (%)	Child n (%)
**Demographic**			
Gender	Female	41 (100)	22 (53.7)
Race	Black	28 (68.3)	27 (65.9)
	White	9 (22.0)	8 (19.5)
Ethnicity	Non-Hispanic	36 (87.8)	36 (87.8)
**Socioeconomic-behavior factors**			
Marital status	Married	2 (4.9)	*na*
Employment	Employed	24 (58.5)	*na*
Education	Middle school	2 (4.9)	*na*
	High school	25 (61.0)	*na*
	Associate	4 (9.8)	*na*
	≥College	10 (24.4)	*na*
**Medical and oral conditions (Y)**			
Smoking		4 (9.8)	*na*
Emotional condition		21 (51.2)	0 (0)
Diabetes		0 (0)	0 (0)
Hypertension		5 (12.2)	0 (0)
Asthma		2 (4.9)	0 (0)
History of yeast infection		12 (29.3)	16 (39.0)
Diaper rash (Child)		*na*	16 (39.0)
Oral thrush (Child)		*na*	8 (19.5)
Caries		32 (78.0)	10 (33.3) ^#^
**Medication use (Y)**			
Antifungal * (Prenatal)		9 (21.9)	*na*
Antibiotic * (Prenatal)		18 (43.9)	*na*
Antifungal * (6 months postpartum or child by 24 m)		5 (12.2)	16 (39.0)
Antibiotic * (6 months postpartum or child by 24 m)		11 (26.8)	10 (24.4)
Inhaler		2 (4.9)	1 (2.4)
**Oral hygiene practice**	Brushing twice daily	28 (68.3)	16 (53.3)
**Birth route**	Vaginal	*na*	32 (78.0)

*na*—not applicable. * Includes any oral suspension, cream/ointments/suppository, or shampoo. ^#^ A total of 33.3% out of the 30 children who completed the study (finished 24 months of follow-up or had caries before 24 months).

**Table 2:** Minimal inhibition concentration (μg/mL) of nystatin, fluconazole, and caspofungin against oral *Candida* isolates, determined by CLSI broth microdilution method.

Species (n)	Antifungal Agent	MIC (μg/mL)
		Range
*Candida albicans* (n = 114)	Nystatin	0.25–8
	Fluconazole	0.25–1
	Caspofungin	0.016–0.25
*Candida parapsilosis* (n = 6)	Nystatin	0.5–4
	Fluconazole	2–4
	Caspofungin	0.25–0.5
*Candida dubliniensis* (n = 4)	Nystatin	0.5
	Fluconazole	0.5
	Caspofungin	0.06–0.12
*Candida lusitaniae* (n = 2)	Nystatin	2
	Fluconazole	1
	Caspofungin	0.03–0.12

MIC—minimum inhibitory concentration. Values averaged for triplicate measurements.

**Table 3:** Susceptibility of clinical isolates to nystatin, interpreted according to CLSI guidelines.

Species and Child Age at Isolation (Number of Isolates)	Susceptible (S) MIC ≤ 2 μg/mL * n (%)	Resistant (R) MIC > 2 μg/mL * n (%)
*Candida albicans*		
Prenatal (36)	33 (91.7)	3 (8.3)
1 month (6)	6 (100)	0 (0)
2 months (14)	14 (100)	0 (0)
4 months (8)	7 (87.5)	1 (12.5)
6 months (11)	11 (100)	0 (0)
12 months (16)	16 (100)	0 (0)
18 months (13)	12 (92.3)	1 (7.7)
24 months (10)	9 (90)	1 (10)
*Candida parapsilosis*		
Prenatal (3)	2 (66.7)	1 (33.3)
4 months (1)	1 (100)	0 (0)
12 months (2)	2 (100)	0 (0)
*Candida dubliniensis*		
Prenatal (2)	2 (100)	0 (0)
24 months (2)	2 (100)	0 (0)
*Candida lusitaniae*		
Prenatal (1)	1 (100)	0 (0)
24 months (1)	1 (100)	0 (0)

MIC—minimum inhibitory concentration. Values averaged for triplicate measurements. * No clinical breakpoints (CBP) or epidemiologic cut-ofAf values (ECV) had been identified for nystatin by CLSI. As an alternative, ECV for amphotericin B has been used as a cut-off value to separate susceptible and resistant isolates [48].

**Table 4:** Susceptibility of clinical *C. albicans* and *C. parapsilosis* to fluconazole, according to CLSI guidelines.

Child Age (Number of Isolates)	Susceptible (S) MIC ≤ 2 μg/mL * n (%)	Susceptible Dose-Dependent (SDD) MIC = 4 μg/mL * n (%)	Resistant (R) MIC ≥ 8 μg/mL * n (%)
*Candida albicans*			
Prenatal (36)	36 (100)	0 (0)	0 (0)
1 month (6)	6 (100)	0 (0)	0 (0)
2 months (14)	14 (100)	0 (0)	0 (0)
4 months (8)	8 (100)	0 (0)	0 (0)
6 months (11)	11 (100)	0 (0)	0 (0)
12 months (16)	16 (100)	0 (0)	0 (0)
18 months (13)	13 (100)	0 (0)	0 (0)
24 months (10)	10 (100)	0 (0)	0 (0)
*Candida parapsilosis*			
Prenatal (3)	2 (66.7)	1 (33.3)	0 (0)
4 months (1)	1 (100)	0 (0)	0 (0)
12 months (2)	1 (50)	1 (50)	0 (0)

* Data presented using clinical breakpoints. MIC—minimum inhibitory concentration. Values averaged for triplicate measurements.

**Table 5:** Susceptibility of clinical *C. albicans* to caspofungin according to CLSI guidelines.

Species and Isolation Timepoint (Number of *Candida albicans* Isolates)	Susceptible (S) MIC ≤ 0.25 μg/mL * n (%)	Intermediate (I) MIC = 0.5 μg/mL * n (%)	Resistant MIC ≥ 1 μg/mL * n (%)
Prenatal (36)	36 (100)	0 (0)	0 (0)
1 month (6)	6 (100)	0 (0)	0 (0)
2 months (14)	14 (100)	0 (0)	0 (0)
4 months (8)	8 (100)	0 (0)	0 (0)
6 months (11)	11 (100)	0 (0)	0 (0)
12 months (16)	16 (100)	0 (0)	0 (0)
18 months (13)	13 (100)	0 (0)	0 (0)
24 months (10)	10 (100)	0 (0)	0 (0)

* Data presented using clinical breakpoints. MIC—minimum inhibitory concentration. Values averaged for triplicate measurements.

**Table 6:** List of missense mutations conserved among different *C. albicans* clinical isolates.

Groups Based on Susceptibility	Gene	Gene Length	Gene Position	Nucleotide Substitution	Aminoacid Substituition	Allele
Nystatin resistance (MIC > 2 μg/mL)	*CDR2*	4500	chr 3	2048G > A	Arg683Lys	homo/hetero
	*CDR2*	4500	chr 3	4011G > C	Leu1337Phe	homo/hetero
Caspofungin reduced susceptibility (MIC = 0.25 μg/mL)	*FKS1*	5694	chr 1	5657C > G	Ser1886Thr	homo/hetero
	*CDR2*	4500	chr 3	2048G > A	Arg683Lys	homo/hetero
	*CDR2*	4500	chr 3	4011G > C	Leu1337Phe	homo/hetero
	*ERG11*	1587	chr 5	348T > A	Glu116Asp	homo/hetero

## Data Availability

Data may be accessed by contacting the corresponding authors.

## Supplementary Materials

The following supporting information can be downloaded at: https://www.mdpi.com/article/10.3390/jof9050580/s1, Table S1. Medical conditions and medication use characteristics of 41 mother-child dyads; Table S2. Classification of clinical oral *C. dubliniensis* isolates susceptibility to fluconazole according to CLSI guidelines using epidemiological cutoff values (ECV); Table S3. Classification of clinical oral *C. lusitaniae* isolates susceptibility to fluconazole according to CLSI guidelines using epidemiological cutoff values (ECV); Table S4. Classification of clinical oral *C. parapsilosis* susceptibility to caspofungin according to CLSI guidelines using clinical breakpoints (CBP); Table S5. Classification of clinical oral *C. dubliniensis* isolates susceptibility to caspofungin according to CLSI guidelines using epidemiological cutoff values (ECV); Table S6. Classification of clinical oral *C. lusitaniae* isolates susceptibility to caspofungin according to CLSI guidelines using epidemiological cutoff values (ECV); Table S7. List of mutations conserved among nystatin-resistant *C. albicans* strains (MIC > 2 μg/mL; n = 6); Table S8. List of mutations conserved among fluconazole wild-type *C. albicans* strains (MIC ≤ 0.5 μg/mL; n = 60); Table S9. List of mutations conserved among caspofungin borderline high MIC values (MIC = 0.25 μg/mL) *C. albicans* strains (n = 7); Figure S1. Proportions of child feeding (breast, bottle, and both breast and bottle feeding), night breast and bottle feeding, solid food intake, daycare attendance, mother and father as care provider measured at 1-, 2-, 4-, 6-, 12-, 18-, and 24-months; Figure S2. Distribution of antifungal medication minimal inhibitory concentration (MIC) values of 4 *C. dubliniensis* clinical isolates from mother-child dyads; Figure S3. Distribution of antifungal medication minimal inhibitory concentration (MIC) values of 6 *C. parapsilosis* clinical isolates from mother-child dyads; Figure S4. Heatmaps representing the relative growth of *Candida* isolates in the presence of different drug concentrations.
