# Supplementary material for: Antifungal Susceptibility of Oral Candida Isolates from Mother-Infant Dyads to Nystatin, Fluconazole, and Caspofungin
Source: J Fungi (Basel). 2023 May 17;9(5):580. doi: 10.3390/jof9050580 (PMC10219145; doi:10.3390/jof9050580)
Supplement: Supplementary file 1 [file jof-09-00580-s001.zip › jof-2385359-supplementary.pdf]

Supplemental Tables and Figures

Table S1. Medical conditions and medication use characteristics of 41 mother-child dyads.

**Category**		**Mother n (%)**	**Child n (%)**
**Mother medication use (Y)**	*Antifungal during pregnancy	9 (21.9)	
	Oral antifungal during pregnancy	6 (14.6)	
	*Antifungal 6m after pregnancy	5 (12.2)	
	Oral antifungal 6m after pregnancy	3 (7.3)	
	*Antibiotic during pregnancy	18 (43.9)	
	Oral antibiotics during pregnancy	16 (39.0)	
	*Antibiotics 6m after pregnancy	11 (26.8)	
	Oral antibiotics 6m after pregnancy	9 (22.0)	
**Child medical conditions/medication (Y)**	Nasal congestion		4 (9.8)
	Otitis media		12 (29.3)
	1st Viral URI		17 (41.5)
	2nd Viral URI		7 (17.1)
	Pharyngitis		2 (4.9)
	Cough		11 (26.8)
	Multiple vomiting, diarrhea constipation		15 (36.6)
	Covid-19		3 (7.3)
	Diaper rash		16 (39)
	Oral thrush		8 (19.5)
	Eczema		8 (19.5)
	Poor weight gain		4 (9.8)
	Viral illness		5 (12.2)
	Abnormal hemoglobin		9 (22)
	High lead		6 (14.6)
	Oral amoxicillin		10 (24.4)
	Oral Nystatin		7 (17.1)
	Nystatin cream/ointment		8 (19.5)
	Oral fluconazole		1 (2.4)
	Clotrimazole cream		3 (7.3)

*Includes any oral suspension, cream/ointments/suppository, or shampoo. URI: upper respiratory infection.

Table S2. Classification of clinical oral *C. dubliniensis* isolates susceptibility to fluconazole according to CLSI guidelines using epidemiological cutoff values (ECV).

Species and Isolation timepoint (number of isolates)	Susceptible (S) MIC ≤0.5 μg/ml n (%)	Resistant (R) MIC >0.5 μg/ml n (%)
Prenatal (2)	2 (100)	0 (0)
24 months (2)	2 (100)	0 (0)

MIC: minimum inhibitory concentration. Values averaged for triplicate measurements. No clinical breakpoints were established for *C. dubliniensis*; alternatively, the ECV was used as a cut-off value to separate susceptible and resistant isolates.

Table S3. Classification of clinical oral *C. lusitaniae* isolates susceptibility to fluconazole according to CLSI guidelines using epidemiological cutoff values (ECV).

Species and Isolation timepoint (number of isolates)	Susceptible (S) MIC ≤2 μg/ml n (%)	Resistant (R) MIC 2μg/ml n (%)
Prenatal (1)	1 (100)	0 (0)
24 months (1)	1 (100)	0 (0)

MIC: minimum inhibitory concentration. Values averaged for triplicate measurements. No clinical breakpoints were established for *C. lusitaniae*; alternatively, the ECV was used as a cut-off value to separate susceptible and resistant isolates.

Table S4. Classification of clinical oral *C. parapsilosis* susceptibility to caspofungin according to CLSI guidelines using clinical breakpoints (CBP).

Timepoint (Number of isolates)	Susceptible (S) MIC ≤2 µg/ml n (%)	Intermediate (I) MIC =4 µg/ml n (%)	Resistant MIC ≥8 µg/ml n (%)
Prenatal (3)	3 (100)	0 (0)	0 (0)
4 months (1)	1 (100)	0 (0)	0 (0)
12 months (2)	2 (100)	0 (0)	0 (0)

MIC: minimum inhibitory concentration. Values averaged for triplicate measurements.

Table S5. Classification of clinical oral *C. dubliniensis* isolates susceptibility to caspofungin according to CLSI guidelines using epidemiological cutoff values (ECV).

Species and Isolation timepoint (number of isolates)	Susceptible (S) MIC ≤0.12 µg/ml n (%)	Resistant (R) MIC >0.12 µg/ml n (%)
Prenatal (2)	2 (100)	0 (0)
24 months (2)	2 (100)	0 (0)

MIC: minimum inhibitory concentration. Values averaged for triplicate measurements. No clinical breakpoints were established for *C. dubliniensis*; alternatively, the ECV was used as a cut-off value to separate susceptible and resistant isolates.

Table S6. Classification of clinical oral *C. lusitaniae* isolates susceptibility to caspofungin according to CLSI guidelines using epidemiological cutoff values (ECV).

Species and Isolation timepoint (number of isolates)	Susceptible (S) MIC ≤1 µg/ml n (%)	Resistant (R) MIC 1µg/ml n (%)
Prenatal (1)	1 (100)	0 (0)
24 months (1)	1 (100)	0 (0)

MIC: minimum inhibitory concentration. Values averaged for triplicate measurements. No clinical breakpoints were established for *C. lusitaniae*; alternatively, the ECV was used as a cut-off value to separate susceptible and resistant isolates.

Table S7. List of mutations conserved among nystatin-resistant *C. albicans* strains (MIC> 2 µg/ml; n=6).

Gene	Gene length	Gene position	Nucleotide substitution	Aminoacid substituition	Allele	Mutation type
FKS1	5694	chr 1	2955A>G		hetero	synonymous mutation
FKS1	5694	chr 1	1929T>A		homo/hetero	synonymous mutation
FKS1	5694	chr 1	1065A>G		homo/hetero	synonymous mutation
FKS1	5694	chr 1	756T>C		homo/hetero	synonymous mutation
CDR2	4500	chr 3	531A>T		homo/hetero	synonymous mutation
CDR2	4500	chr 3	1572C>T		homo/hetero	synonymous mutation
CDR2	4500	chr 3	1894C>T		homo/hetero	synonymous mutation
CDR2	4500	chr 3	2048G>A	Arg683Lys	homo/hetero	missense mutation
CDR2	4500	chr 3	2169T>C		homo/hetero	synonymous mutation
CDR2	4500	chr 3	4011G>C	Leu1337Phe	homo/hetero	missense mutation
CDR1	4506	chr 3	69T>C		homo/hetero	synonymous mutation
CDR1	4506	chr 3	423A>T		homo/hetero	synonymous mutation
CDR1	4506	chr 3	435C>T		homo/hetero	synonymous mutation
CDR1	4506	chr 3	537A>T		homo/hetero	synonymous mutation
CDR1	4506	chr 3	663C>T		hetero	synonymous mutation
CDR1	4506	chr 3	2712A>G		homo/hetero	synonymous mutation
CDR1	4506	chr 3	3225A>G		homo/hetero	synonymous mutation
CDR1	4506	chr 3	3237A>T		homo/hetero	synonymous mutation
CDR1	4506	chr 3	3267G>A		homo/hetero	synonymous mutation
CDR1	4506	chr 3	3660T>C		homo/hetero	synonymous mutation
CDR1	4506	chr 3	3717C>T		homo/hetero	synonymous mutation
MDR1	1695	chr 6	1041A>G		homo/hetero	synonymous mutation
MDR1	1695	chr 6	909C>T		homo/hetero	synonymous mutation
MDR1	1695	chr 6	843A>G		homo/hetero	synonymous mutation
MDR1	1695	chr 6	645A>G		homo/hetero	synonymous mutation
MDR1	1695	chr 6	624A>G		homo/hetero	synonymous mutation
MDR1	1695	chr 6	612A>T		homo/hetero	synonymous mutation
MDR1	1695	chr 6	597A>G		homo/hetero	synonymous mutation
MDR1	1695	chr 6	471T>A		homo/hetero	synonymous mutation
MDR1	1695	chr 6	468C>T		homo/hetero	synonymous mutation

Table S8. List of mutations conserved among fluconazole wild-type *C. albicans* strains (MIC≤ 0.5 µg/ml; n=60).

Gene	Gene length	Gene position	Nucleotide substitution	Aminoacid substituition	Allele	Mutation type
FKS1	5694	chr 1	1065A>G		homo/hetero	synonymous mutation
CDR1	4506	chr 3	3717C>T		homo/hetero	synonymous mutation
MDR1	1695	chr 6	1308G>A		homo/hetero	synonymous mutation
MDR1	1695	chr 6	1287T>C		homo/hetero	synonymous mutation
MDR1	1695	chr 6	1272T>C		homo/hetero	synonymous mutation

Table S9. List of mutations conserved among caspofungin borderline high MIC values (MIC= 0.25 μg/ml) *C. albicans* strains (n=7).

Gene	Gene length	Gene position	Nucleotide substitution	Aminoacid substituition	Allele	Mutation type
FKS1	5694	chr 1	5657C>G	Ser1886Thr	homo/hetero	missense mutation
FKS1	5694	chr 1	4230A>G		homo/hetero	synonymous mutation
FKS1	5694	chr 1	1359G>A		homo/hetero	synonymous mutation
FKS1	5694	chr 1	1350A>G		homo/hetero	synonymous mutation
FKS1	5694	chr 1	1065A>G		homo/hetero	synonymous mutation
CDR2	4500	chr 3	531A>T		homo/hetero	synonymous mutation
CDR2	4500	chr 3	1527C>T		homo/hetero	synonymous mutation
CDR2	4500	chr 3	1894C>T		homo/hetero	synonymous mutation
CDR2	4500	chr 3	2048G>A	Arr683Lys	homo/hetero	missense mutation
CDR2	4500	chr 3	2169T>C		homo/hetero	synonymous mutation
CDR2	4500	chr 3	4011G>C	Leu1337Phe	homo/hetero	missense mutation
CDR2	4500	chr 3	4410T>C		homo/hetero	synonymous mutation
CDR1	4506	chr 3	423A>T		homo/hetero	synonymous mutation
CDR1	4506	chr 3	435C>T		homo/hetero	synonymous mutation
CDR1	4506	chr 3	438A>T		homo/hetero	synonymous mutation
CDR1	4506	chr 3	3225A>G		hetero	synonymous mutation
CDR1	4506	chr 3	3237A>T		hetero	synonymous mutation
CDR1	4506	chr 3	3267G>A		homo/hetero	synonymous mutation
ERG11	1587	chr 5	1470G>A		homo/hetero	synonymous mutation
ERG11	1587	chr 5	658G>A		hetero	synonymous mutation
ERG11	1587	chr 5	411A>G		homo/hetero	synonymous mutation
ERG11	1587	chr 5	357C>T		homo/hetero	synonymous mutation
ERG11	1587	chr 5	348T>A	Glu116Asp	homo/hetero	missense mutation
MDR1	1695	chr 6	1308G>A		homo/hetero	synonymous mutation
MDR1	1695	chr 6	1287T>C		homo/hetero	synonymous mutation
MDR1	1695	chr 6	1272T>C		homo/hetero	synonymous mutation
MDR1	1695	chr 6	1041A>G		homo/hetero	synonymous mutation
MDR1	1695	chr 6	645A>G		homo/hetero	synonymous mutation
MDR1	1695	chr 6	624A>G		homo/hetero	synonymous mutation
MDR1	1695	chr 6	612A>T		homo/hetero	synonymous mutation
MDR1	1695	chr 6	597A>G		homo/hetero	synonymous mutation
MDR1	1695	chr 6	471T>A		homo/hetero	synonymous mutation
MDR1	1695	chr 6	468C>T		homo/hetero	synonymous mutation

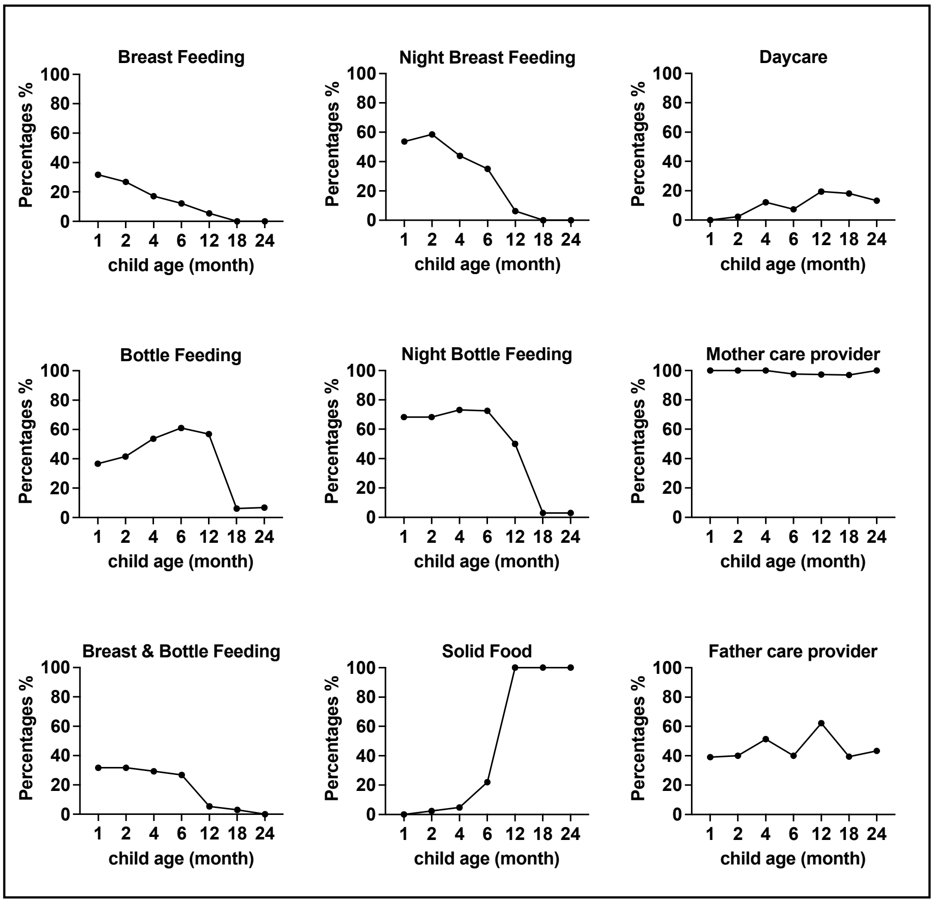

Figure S1. Proportions of child feeding (breast, bottle, and both breast and bottle feeding), night breast and bottle feeding, solid food intake, daycare attendance, mother and father as care provider measured at 1-, 2-, 4-, 6-, 12-, 18-, and 24-months. The feeding patterns, including breastfeeding, bottle feeding (exclusively), night feeding, and consumption of solid food, daycare attendance and care provider are illustrated. Exclusively, breastfeeding gradually decreased from 32% at one month to 0% at 18 months. On the other hand, exclusively bottle feeding nearly doubled from 36% at one month to 61% at six months, remained stable between six to 12 months, and sharply decreased at 18 months. The breast and bottle-fed portion remained stable at approximately 30% from one to six months, then reduced to 0% at 24 months. Night breastfeeding was high during the first two months (58%), gradually decreasing as the children got older. Moreover, children who had night bottle feeding were high (73%) during the first six months, with a sharp drop after six months, reaching 3% at the age of two years. Consumption of solid food started as early as two months and peaked at 100% at 12 months. Daycare attendance was low and remained below 20% for all study points. Mother was involved in the care of their child approximately 100% of the time. However, the father was involved in the care of his/her child between 39% - 62% of the time.

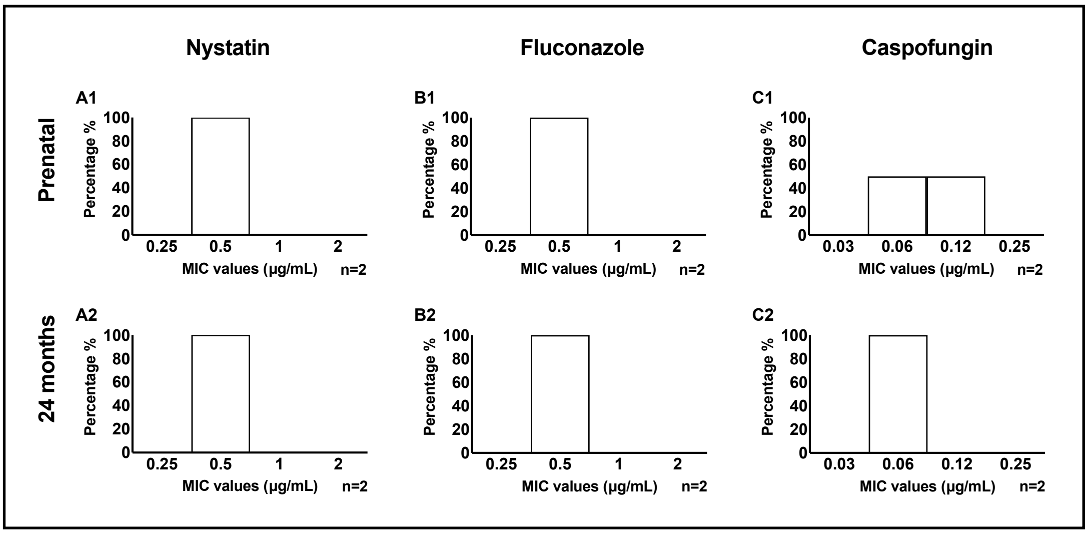

Figure S2. Distribution of MIC values of three antifungal drugs tested in four *C. dubliniensis* clinical isolates from mother-child dyads**.** The MIC values for nystatin, fluconazole, and caspofungin are illustrated separately in A1-3, B1-3, and C1-3. For nystatin and fluconazole, 100% of the mothers' and children's isolates had MIC values of 0.5 µg/ml (A1-B2). For caspofungin, all the isolates had MIC value of 0.06 except one mother's isolate had a value of 0.12 µg/ml (C1-3).

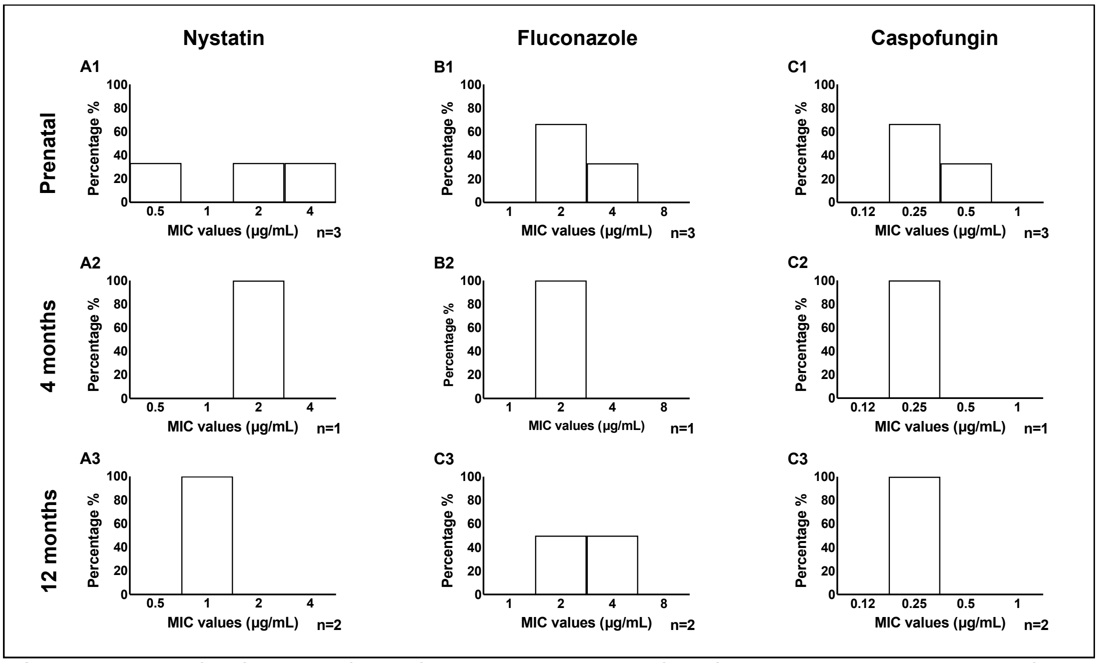

Figure S3. Distribution of MIC values of 3 antifungal drugs tested in of six *C. parapsilosis* clinical isolates from mother-child dyads. The MIC values for nystatin, fluconazole, and caspofungin are illustrated in A1-3, B1-3, and C1-3 separately. For nystatin (A1-3), each one of the mother isolates had a different MIC value ranging between 0.5 - 4 µg/ml (A1). All children's *C. parapsilosis* isolates had MIC values ≤2 µg/ml (A2-3). Regarding fluconazole (B1-3), mothers and children had the same frequency distribution of MIC values centered around 2-4 µg/ml. For caspofungin (C1-3), all the isolates had a MIC value of 0.25 µg/ml except one mother's isolate, with a higher value of 0.5 µg/ml.

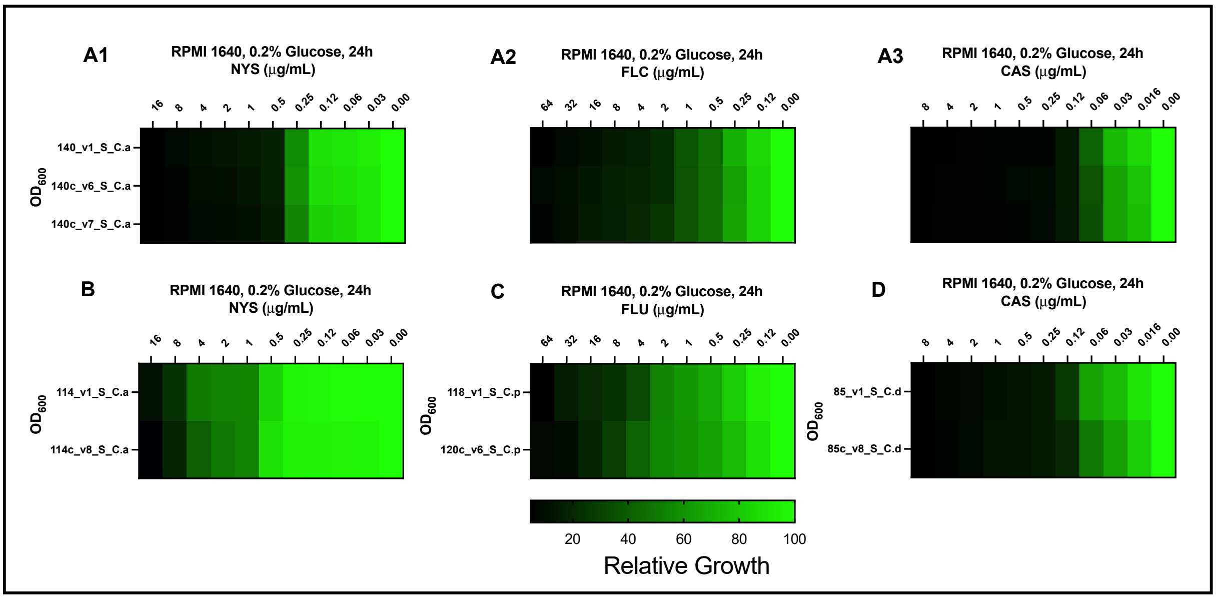

Figure S4. Heatmaps representing the relative growth of each isolate in the presence of different drug concentrations. MIC assays were performed in RPMI medium in the presence of NYS, FLC, or CAS. OD_{600} was measured after 24 h, and the values were normalized to the no-drug (growth) controls. Data were displayed using GraphPad Prism version 9.4.1. see the color bar representing the relative growth. Abbreviations; RPMI: Roswell Park Memorial Institute Medium, NYS: nystatin, FLC: fluconazole, CAS: caspofungin, MIC: minimum inhibitory concentration, OD: optical density, v: study visit number, S: saliva, C.a: *C. albicans*, C.d: *C. dubliniensis*, C.p: *C. parapsilosis*. Example of one mother-child dyad with *C. albicans* displaying the same MIC values for the mother and the two visits for the child for all tested drugs; this also indicates no change in the child's MIC values over time (A1-3). Cases of nystatin resistance where *C. albicans* from mom-child dyad had MIC values >2 µg/ml (B). Situations of susceptible dose-dependent of *C. parapsilosis* to fluconazole, MIC= 4 µg/ml (C). A pair with *C. dubliniensis*, where the child's isolate had a lower MIC value for caspofungin, MIC=0.06 µg/ml, compared to the mother's isolate, MIC= 0.12 µg/ml (D).
